# Synthesis of Thermo-Responsive Monofunctionalized Diblock Copolymer Worms

**DOI:** 10.3390/polym15234590

**Published:** 2023-11-30

**Authors:** Xuan Xue, Feifei Wang, Minhao Shi, Faez Iqbal Khan

**Affiliations:** 1 Department of Chemistry, School of Science, Xi’an Jiaotong-Liverpool University, Suzhou 215123, China; feifei.wang22@student.xjtlu.edu.cn (F.W.); minhao.shi15@student.xjtlu.edu.cn (M.S.); 2 Department of Biological Sciences, School of Science, Xi’an Jiaotong-Liverpool University, Suzhou 215123, China; faez.khan@xjtlu.edu.cn

**Keywords:** PGMA-PHPMA, diblock copolymer worm, worm gel, thermo-responsive hydrogel, post-modification, monofunctionalization, RAFT, PISA, bioconjugation

## Abstract

Poly(glycerol monomethacrylate)-*block*-poly(2-hydroxypropyl methacrylate) (PGMA-PHPMA) with worm-like morphology is a typical example of reversible addition–fragmentation chain transfer (RAFT) dispersion polymerized thermo-responsive copolymer via polymerization-induced self-assembly (PISA) in aqueous solution. Chain transfer agents (CTAs) are the key component in controlling RAFT, the structures of which determine the end functional groups of the polymer chain. It is therefore of interest to monofunctionalize the polymers via CTA moiety, for bioactive functionality conjugation and in the meantime maintain the precisely controlled morphology of the copolymers and the related property. In this work, a newly designed CTA 5-(2-(*tert*-butoxycarbonylamino) ethylamino)-2-cyano-5-oxopentan-2-yl benzodithioate *(t-Boc* CPDB) was synthesized and used for the RAFT polymerization of PGMA_45_-PHPMA_120_. Subsequently, PGMA_45_-PHPMA_120_ copolymers with primary amine, maleimide, and reduced L-glutathione (a tripeptide) monofunctionalized terminals were synthesized via deprotection and conjugation reactions. These monofunctionalized copolymers maintain worm-like morphology and thermo-responsive property in aqueous solution (10% *w*/*v*), as confirmed by the transmission electron microscopy (TEM) images, and the observation of the phase transition behavior in between 4 °C and room temperature (~20 °C), respectively. Summarily, a range of thermo-responsive monofunctionalized PGMA_45_-PHPMA_120_ diblock copolymer worms were successfully synthesized, which are expected to offer potential biomedical applications, such as in polymer therapeutics, drug delivery, and diagnostics.

## 1. Introduction

Reversible addition–fragmentation chain transfer (RAFT) polymerization is one of the most popularly used controlled living radical polymerization methods due to its applicability to a wide range of monomers and solvents [1]. Different from other controlled polymerization approaches where metal catalysts are needed, chain transfer agents (CTAs) are used to mediate the chain transfer equilibrium of the RAFT process, and to achieve the determined molecular weight (Mw) and low polydispersity (PDI). A general RAFT CTA formula is Z-C(=S)=S-R, where R group influences reactivity of dithiocarbonyl carbon and reinitiates polymerization and Z group modulates reactivity of dithiocarbonyl carbon and stabilizes intermediate radical. Upon termination, R and Z groups are bound at the end of the polymer chain, where the creation of more functional groups at either end is possible [2]. This is particularly useful when a bioactive functionality is to be conjugated to the end of a polymer [3,4].

Nowadays, an increasing interest is shown in the synthesis of primary amine-containing polymers for their wide biological applications in gene carriers [5,6], antimicrobial coatings [7,8], and bioconjugations [9,10]. One of the most popular primary amine-containing polymers that was reported is the commercially available 2-aminoethyl methacrylate hydrochloride (AMA)-based polymer [11,12], while the chemical degradation of PAMA was observed due to the nucleophilic nature of primary amine in alkaline media [13]. Therefore, *tert*-butoxycarbonyl (*t*-*Boc*) protection of amine was employed for the RAFT polymerization of 2-aminoethyl methacrylate [14]. In particular, the same protection and deprotection strategy is also applicable before and after RAFT polymerization when a primary amine moiety is in the CTA structure [15]. Indeed, it is of interest to generate mono-aminated polymer with potential pH-responsive properties and conjugation reactivity, which simultaneously maintains the precisely controlled morphology and desirable characteristics (e.g., thermo-responsive) for biological applications.

The use of polymerization-induced self-assembly (PISA) to produce various copolymer nano-architectures via either RAFT dispersion or emulsion polymerization is widespread among the polymer chemists [16,17,18], due to the versatile and highly efficient process compared to the traditional post-polymerization approach performed in highly dilute solution (<1%) [19]. Briefly, this technique allows for the simultaneous polymerization of well-defined block copolymers and the formation of nano-architectures (e.g., spherical micelles, worm-like micelles or vesicles) to be conducted, particularly, with relatively high concentration (10–25%, *w*/*w*) in aqueous solution. Poly(glycerol monomethacrylate) (PGMA) as the stabilizer block and thermo-responsive poly (2-hydroxypropyl methacrylate) (PHPMA) as the core forming block are typically used in formulation, which was well studied [16,20,21]. It is therefore predictive to target desired nano-architecture consistently referring to the complete PGMA-PHPMA diblock copolymer phase diagram [20]; for example, the synthesis of PGMA-PHPMA diblock copolymer with the determined monomer composition and solid concentration, and to target the typical soft free-standing physical hydrogels with worm-like morphology at room temperature [21,22].

Physical hydrogels with self-assembled worm-like morphology exhibit attractive behaviors in cell entry and circulation time compared to the other nano-architectures [23,24]. It was also reported that the biocompatible hydrogels offered a wide range of biological applications, such as tissue engineering scaffold [25,26,27], contact lenses [28,29,30], and gene/drug delivery [31,32,33]. Among them, the thermo-responsive PGMA-PHPMA worm gel, which can be easily sterilized via ultra-filtration after cooling as temperature triggers worm-to-sphere phase transition [22], offers a critical feature for biomedical applications. For example, PGMA-PHPMA worm gel was reported as a bioinert 3D matrix for pluripotent stem cell (PSC) and human embryos storage without cryopreservation [34]. In another work, PGMA-PHPMA worm gels combined with poly(viny alcohol) (PVA) were conducted as an effective cryo-preservative for red blood cells without involving any toxic organic solvents, where the cryopreservation mixture formed free-standing gels upon warming up to room temperature [35]. Moreover, disulfide functionalized PGMA-PHPMA-based worm gels provided improved mechanical stability as a 3D matrix for long-term A549 human lung cancer cell culture. This system offered good cell viability and facilitated a cell recovery process simply by incubating at a lowered temperature (4 °C) [36]. More recently, PGMA-PHPMA worm gel was explored as an injectable cell delivery scaffold, which directed human adipose-derived mesenchymal stem cells to differentiate into nucleus pulposus cells, for treating degenerate disk disease [37].

To the best of our knowledge, the use of PISA formulation via RAFT polymerization to generate well-defined PGMA-PHPMA worms, which are subsequently modified to give a series of monofunctionalized terminals on polymer, was not reported. Post-decorated hydrogels are ideally expected to offer combined properties from both the PGMA-PHPMA worms and the surface functionalities, thereby providing expanded biomedical applications.

Here, the novel-designed CTA 5-(2-(*tert*-butoxycarbonylamino)ethylamino)-2-cyano-5-oxopentan-2-yl benzodithioate (*t-Boc* CPDB) was synthesized from the commercial available 4-Cyano-4-(phenylcarbonothioylthio)pentanoic acid (CPADB) via a high-yielding two-step reaction involving a succinimide ester-activated intermediate. PGMA_45_ was polymerized using *t-Boc* CPDB as a chain transfer agent (CTA) via the RAFT polymerization method. HPMA was then RAFT aqueous dispersion polymerized at a copolymer concentration of 10 *w*/*v*% to produce well-defined block copolymers using *t-Boc*-PGMA_45_ as macro-CTA, targeting worm-like micelles in terms of the reported phase diagram [20]. The aminated PGMA_45_-PHPMA_120_ was then obtained via deprotection of the *t*-*Boc* group from the amine. The conjugation synthesis subsequently started from maleimide functionalization via the primary amine groups to the end of the polymer, for maleimide is known as a linker for peptide conjugation that can rapidly react with thiol by a Michael addition in mild aqueous condition [38,39]. Reduced L-glutathione (GSH) was conducted as a model thiol-containing tripeptide and conjugated by reacting with maleimide terminal. All the synthesized PGMA_45_-PHPMA_120_ diblock copolymers were characterized with proton nuclear magnetic resonance (^1^H-NMR) and gel permeation chromatography (GPC). The copolymer solids were re-dispersed at 10 *w*/*v*% in PBS (100 mM, pH 7.4) at room temperature and re-form free-standing gels [40], which became liquid at 4 °C due to temperature-triggered worm-to-sphere phase transition. The zeta-potential measurements of *t*-*Boc*, aminated, and maleimide-functionalized PGMA_45_-PHPMA_120_ copolymer worms were performed using dynamic light scattering (DLS) at pH values ranging from 2 to 10, where positive surface charge was observed at low pH due to amine protonation. The maintenance of worm-like morphology before and after the multi-step post-polymerization modifications was confirmed using transmission electron microscopy (TEM). Here, monofunctionalized PGMA_45_-PHPMA_120_ copolymer worms successfully synthesized with reversible thermo-responsive behavior are expected to offer potential biomedical applications, such as in polymer therapeutics, drug delivery, and diagnostics.

## 2. Materials and Methods

### 2.1. Materials

2-Hydroxypropyl methacrylate (HPMA, 97%), 4,4′-Azobis(4-cyanovaleric acid) (ACVA, V-501; 99%), 4-cyano-4-(phenylcarbonothioylthio)pentanoic acid (CPADB; 97%), *N*-hydroxysuccinimide (NHS; 98%), dicyclohexylcarbodiimide (DCC; 99%), *N*-Boc-ethylenediamine (NBEDA; ≥98%), proton sponge (99%), 3-maleimidopropionic acid *N*-hydroxysuccinimide ester (MPA-NHS; 99%), L-glutathione reduced (GSH; ≥98%), and spectra-Por dialysis tubing (MWCO 1 kD) were purchased from Merck (Shanghai, China) and used as received. Glycerol monomethacrylate (GMA; 95%) was purchased from Macklin (Shanghai, China) and further purified by running through silica column (eluent: ethyl acetate:hexane = 1:2). Deuterated methanol (CD_3_OD; 99.96 atom%) and deuterated chloroform (CDCl_3_; 99.96 atom%) were purchased from Aladdin (Shanghai, China). Solvents were obtained from Energy Chemical (Shanghai, China) and used as received.

### 2.2. Nuclear Magnetic Resonance (NMR) Spectroscopy

^1^H-NMR and ^13^C-NMR were recorded using a 400 MHz Bruker (Shanghai, China) spectrometer with a 64-average number of scans per spectrum. The unit of all chemical shifts recorded is ppm (δ).

### 2.3. Mass Spectroscopy (MS)

Mass spectra were recorded on a Waters 2795 separation module/micro mass LCT platform, under positive scan mode with direct injection of the purified compounds.

### 2.4. Gel Permeation Chromatography (GPC)

The DMF GPC, which comprised two 5 µm Mixed-C columns; a Varian (Palo Alto, CA, USA) 290-LC pump injection module, and a Varian (Palo Alto, CA, USA) 390-LC refractive index detector, was used to determine polymer molecular weight and polydispersity all through the experiments at 60 °C. The HPLC grade DMF eluent contained 10 mM LiBr at a flow rate of 1.0 mL/min. DMSO (10 μL/mL) was added into sample solutions as a flow rate marker. The GPC calibration was established by using a series of ten near-monodisperse poly(methyl methacrylate) (PMMA) standards (M_n_ ranging from 625 to 618,000 g/mol) in conjunction with the refractive index detector. The data were analyzed using Varian Cirrus (Palo Alto, CA, USA) GPC software (version 3.3).

### 2.5. Transmission Electron Microscopy (TEM)

Copper/palladium TEM grids (agar Scientific, Essex, UK) were surface-coated in-house to generate a thin film of amorphous carbon. The grids were then plasma glow-discharged for 30 s to produce a hydrophilic surface. One drop (11 μL) of sample solution containing the diblock copolymer nano-particles (0.20 *w*/*v*%) was loaded on the glow-discharged for 1 min, and the excess solution was blotted with filter paper. The sample-loaded grid was then negatively stained with uranyl formate (0.75 *w*/*v*%, 9 μL) for 20 s to improve the electron contrast of images. The excess dye was removed again, and the grid was dried with a vacuum hose. Imaging was performed on a FEI Tecnai Spirit TEM instrument (FEI Co., Hillsboro, OR, USA) equipped with a Gatan 1kMS600CW CCD camera at 120 kV (Gatan Inc., Pleasanton, CA, USA).

### 2.6. Dynamic Light Scattering (DLS)

The zeta-potential and electrophoretic mobility measurements were conducted at 25 °C using DLS (Malvern Zetasizer NanoZS instrument, Malvern Panalytical Ltd., Malvern, UK) equipped with laser Doppler micro-electrophoresis, and connected to the MPT-2 autotitrator, enabling the study of the effect of changes in pH. The copolymer solids were dissolved with PBS (100 mM, pH 7.4) to form gels, then diluted 50-fold with KCl (0.001 M). The data reported were averaged over three consecutive runs with standard deviation.

### 2.7. Synthesis of CTA 5-(2-(Tert-Butoxycarbonylamino)ethylamino)-2-cyano-5-oxopentan-2-yl benzo-dithioate, t-Boc CPDB

CPADB (2.48 g, 8.9 mmol) and NHS (1.04 g, 8.9 mmol) were co-dissolved in anhydrous dichloromethane (DCM, 18 mL). DCC (1.83 g, 8.9 mmol) was added to this solution and the reaction mixture was stirred at 20 °C in the dark overnight. Insoluble white solids were removed by filtration. The solvent was removed using a rotary evaporator, and the resulting solid was washed with n-hexane twice and then diethyl ether three times. A red solid product 4-cyano-4-((thiobenzoyl)pentanoic succinimide ester (SCPDB) was obtained after evaporation of the solvent residue (yield: 2.77 g, 83%). Before carrying out the second step reaction, all glassware was rigorously dried at 120 °C overnight to remove all traces of water. SCPDB (1.20 g, 3.2 mmol) was dissolved in anhydrous dichloromethane (10 mL) in a 100 mL two-neck round-bottomed flask fitted with a dropping funnel. A mixed solution of NBEDA (0.56 g, 2.8 mmol) and proton sponge (0.72 g, 3.3 mmol) pre-stirred in anhydrous dichloromethane (34 mL) for 3 h was added dropwise to the SCPDB solution over a period of approximately 1 h. This reaction solution was stirred overnight at room temperature. The solution was concentrated using a rotary evaporator, and the resulting liquid was purified by silica column chromatography using ethyl acetate. A red solid final product was isolated after evaporation of the solvent (yield: 1.09 g, 95%).

### 2.8. Synthesis of t-Boc ethylenediaminated Poly(glycerol methacrylate) (t-Boc-PGMA_45_) Macro-CTA and Purification

In a typical experiment of synthesis of *t-Boc*-PGMA_45_, ACVA initiator (0.04846 g, 0.17 mmol), GMA monomer (9.00 g, 56.19 mmol), and *t-Boc* CPDB (0.3644 g, 0.86 mmol) ([GMA]:[*t-Boc* CPDB]:[ACVA] molar ratio = 65:1:0.2) were weighted into a round-bottomed flask containing a magnetic stir bar. These reagents were dissolved in a previously deoxygenated absolute ethanol (14.12 mL, 40% *w*/*v*) and purged with nitrogen for 30 min. The flask was sealed using a rubber septum under a positive nitrogen flow and immersed in an oil bath at 70 °C. The reaction was quenched by exposure to air and cooling to 20 °C after 90 min. The crude product was purified and precipitated in dichloromethane. The solvent residue was removed under vacuum evaporator (degree of polymerization (DP): 44.6; yield: 5.62 g, 60%).

### 2.9. RAFT Dispersion Polymerization of 2-Hydroxypropyl Methacrylate (HPMA) Using t-Boc-PGMA_45_ Macro-CTA

In a typical experiment of synthesis of *t-Boc*-PGMA_45_-PHPMA_120_ at 10% *w*/*v* solids using *t-Boc*-PGMA_45_ macro-CTA, ACVA initiator (0.0011 g, 0.0037 mmol), *t-Boc*-PGMA macro-CTA (0.0848 g, 0.0112 mmol) ([CTA]:[ACVA] molar ratio = 3.0), and HPMA monomer (0.20 g, 1.35 mmol, target DP = 120) were weighted into a 10 mL vial containing a magnetic stir bar. These reagents were dissolved in previously deoxygenated PBS (2.57 mL, 100 mM, pH 7.4) and purged with nitrogen for 30 min. The vial was sealed using a rubber septum and immersed in an oil bath at 70 °C. The reaction was quenched by exposure to air and cooling to 20 °C after 2 h. The resulting crude product was dialyzed against de-ionized water using a 1 kD MWCO dialysis membrane and then freeze-dried (DP: 120; yield: 0.26 g, 92%).

### 2.10. Synthesis of Amine Monofunctionalized PGMA_45_-PHPMA_120_ (NH_3_Cl-PGMA_45_-PHPMA_120_)

In a typical experiment, *t-Boc*-PGMA_45_-PHPMA_120_ (100 mg) was dissolved in methanol (1 mL), and hydrochloric acid (0.5 mL, 10 M) was added dropwise while cooling in an ice-water bath. The mixture was stirred at room temperature for 4 h. The resulting polymer with protonated primary amine end group was dialyzed against de-ionized water using a 1 kD MWCO dialysis membrane and then freeze-dried (yield: 88 mg, 88%).

### 2.11. Synthesis of Maleimide Monofunctionalized PGMA_45_-PHPMA_120_ (Mal-PGMA_45_-PHPMA_120_)

*NH_3_Cl*-PGMA_45_-PHPMA_120_ (50 mg, 0.0020 mmol) was dissolved in anhydrous DMF (750 μL). MPA-NHS (0.80 mg, 0.0030 mmol) and proton sponge (2.15 mg, 0.0100 mmol) were weighed together, dissolved in anhydrous DMF (250 μL), and added dropwise into the above *NH_3_Cl*-PGMA_45_-PHPMA_120_ DMF solution at room temperature. The reaction solution was stirred at room temperature for 6 h. The resulting mixture was dialyzed with a MWCO 1 kD dialysis membrane against de-ionized water and freeze-dried (yield: 39 mg, 78%).

### 2.12. Conjugation of Reduced L-Glutathione on PGMA_45_-PHPMA_120_ (GSH-PGMA_45_-PHPMA_120_)

*Mal*-PGMA_45_-PHPMA_120_ (50 mg) was weighed and dissolved in a solution of GSH (1.0 mL, 1 mg/mL) in PBS (100 mM, pH 7.4). The above solution was stirred overnight and dialyzed with MWCO 3.5 kD dialysis membrane against de-ionized water for 24 h and then freeze-dried (yield: 37 mg, 74%).

## 3. Results and Discussion

### 3.1. t-Boc CTA Synthesis

**Scheme 1:**
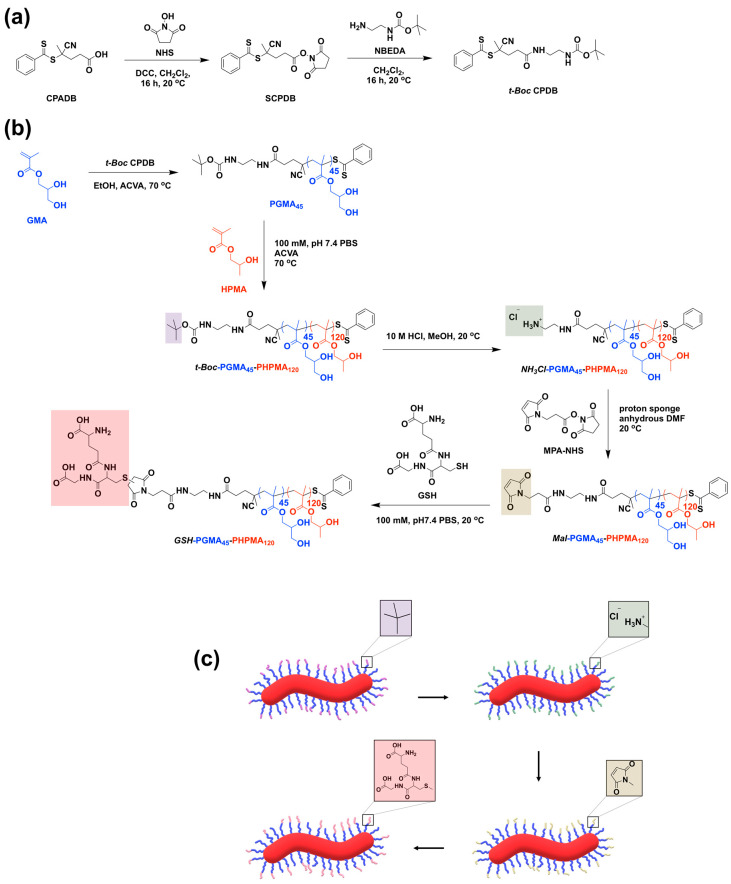
(**a**) Synthesis of *t-Boc* CPDB by a two-step reaction; (**b**) synthesis of PGMA_45_ macro-CTA using *t-Boc* CPDB as CTA; synthesis of *t-Boc*-PGMA_45_-PHPMA_120_, *NH_3_Cl*-PGMA_45_-PHPMA_120_, *Mal*-PGMA_45_-PHPMA_120_, and *GSH*-PGMA_45_-PHPMA_120_ copolymers using RAFT polymerization method and to form diblock copolymer worms either via PISA or via solid re-dispersion in phosphate buffered saline (PBS, 100 mM, pH 7.4, at 10% *w*/*v*); and (**c**) schematic description of the maintenance of worm-like morphology after multi-step post-modifications with mono-functional groups on the surface of the copolymer worms in PBS (100 mM, pH 7.4, at 10% *w*/*v*).

The *t-Boc* CPDB was prepared by a two-step synthesis (Scheme 1a). It was well reported that *N*-hydroxysuccinimide-activated ester of CPADB offers high reactivity to primary amine compared to carboxylic acid. Thus, succinimide-modified cyanopentanoate dithiobenzoate (SCPDB) was synthesized according to a previously reported protocol [41]. The purification steps were modified to simplify the operation procedure via washing the crude products with n-hexane and then diethyl ether. ^1^H-NMR spectroscopy confirmed the chemical structure of the target compound with high purity. Then, SCPDB was reacted with *N*-Boc ethylenediamine in anhydrous DCM overnight to yield *t-Boc* CPDB. The resulting product was efficiently isolated with high yield (95%) after running through silica column (eluent: ethyl acetate). ^1^H-NMR, ^13^C-NMR, HMQC-NMR, and mass spectroscopy were consistent with the target compound (Figures S1 and S2 in ESI).

### 3.2. Macro-CTA Synthesis

**Figure 1:**
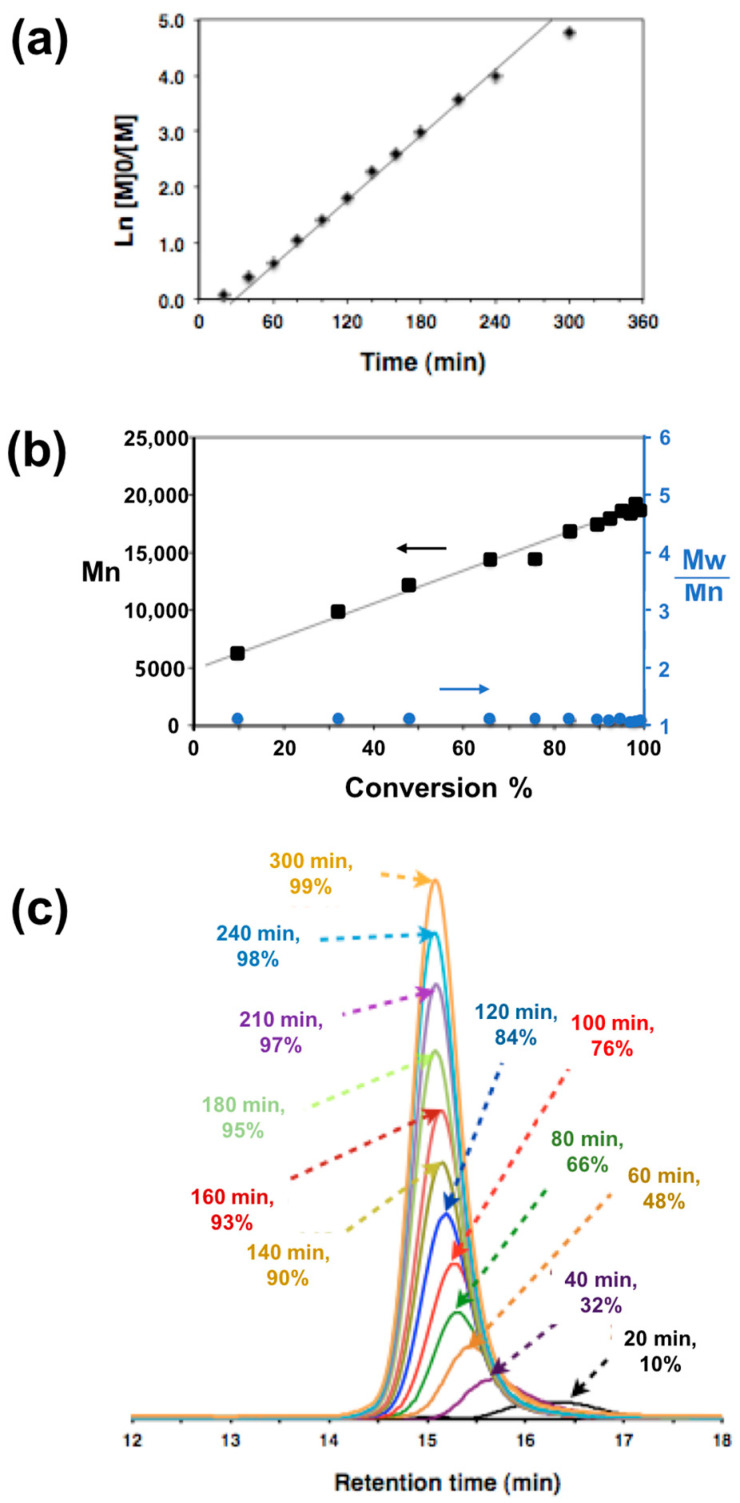
(**a**) Semilogarithmic plot (**b**) Mn (black squares) and PDI (blue circles) as a function of monomer conversion and (**c**) DMF GPC curves indicating the molecular weight evolution with elution time for the RAFT homopolymerization of GMA at 70 °C using *t-Boc* CPDB as CTA in ethanol at a relative molar ration of [GMA]:[*t-Boc* CPDB]:[ACVA] = 65:1.0:0.2.

*t-Boc*-PGMA_45_ macro-CTA was RAFT polymerized in a one-pot protocol according to Scheme 1b following a previously published protocol [22]. PGMA RAFT homopolymerization in ethanol was successful when using *t-Boc* CPDB as the CTA and using ACVA as initiator (molar ratio of *t*-*Boc* CPDB/ACVA = 5.0). Kinetic studies conducted at 70 °C using an initial [GMA]_0_/[*t-Boc* CPDB]_0_ molar ratio (or target DP) of 65 gave a linear semilogarithmic plot indicating first-order kinetics with respect to monomer (Figure 1a). Monomer conversions were calculated by ^1^H-NMR spectroscopy by comparing the integral of vinyl (δ 5.66 and δ 6.16 ppm) to that of methylene and methyne of GMA signals (δ 3.83–4.30 ppm). The DMF GPC data measured from aliquots taken from the GMA homopolymerization indicated that the molecular weight increased in a monotonic manner as a function of conversion and the polydispersity remained relatively narrow throughout the polymerization (Figure 1b). The DMF GPC unimodal traces of aliquots shifted smoothly to a lower elution volume with the increasing monomer conversion, confirming maintained RAFT control (Figure 1c). In all cases, the M_n_ values measured from DMF GPC were slightly higher than those calculated from monomer conversion based on ^1^H-NMR data, which is due to the structural difference between the poly(methyl methacrylate) (PMMA) standards and the *t-Boc*-PGMA_45_ homopolymers [11,42,43]. The *t-Boc*-PGMA_45_ homopolymer macro-CTA was prepared under the above reaction condition, and quenched at 90 min. The monomer conversions of both crude and purified products calculated from ^1^H-NMR in d-methanol were consistently 69%, thus further confirming the high purity of the in-house synthesized *t-Boc* CPDB.

### 3.3. Synthesis of NH_3_Cl-PGMA_45_-PHPMA_120_ with Worm-Like Morphology

**Figure 2:**
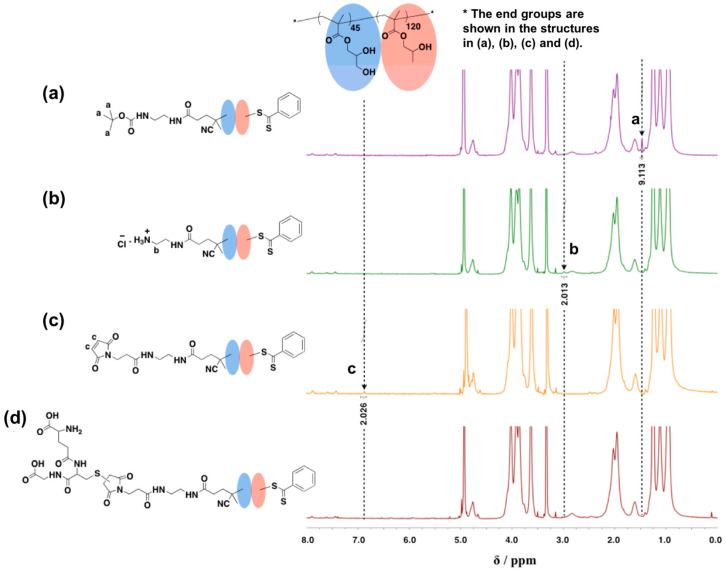
^1^H-NMR (400 Hz, d-methanol) spectra recorded for (**a**) *t*-*Boc* protected PGMA_45_-PHPMA_120_ copolymer RAFT polymerized by using *t-Boc*-PGMA_45_ macro-CTA in PBS (100 mM, pH 7.4), (**b**) *NH_3_Cl*-PGMA_45_-PHPMA_120_ generated with HCl (10 M) in methanol, (**c**) *Mal*-PGMA_45_-PHPMA_120_ copolymer synthesized by reacting *NH_3_Cl*-PGMA_45_-PHPMA_120_ with MPA-NHS in anhydrous DMF at room temperature, and (**d**) GSH conjugated PGMA_45_-PHPMA_120_ copolymer synthesized in 100 mM PBS (100 mM, pH 7.4) at room temperature.

**Figure 3:**
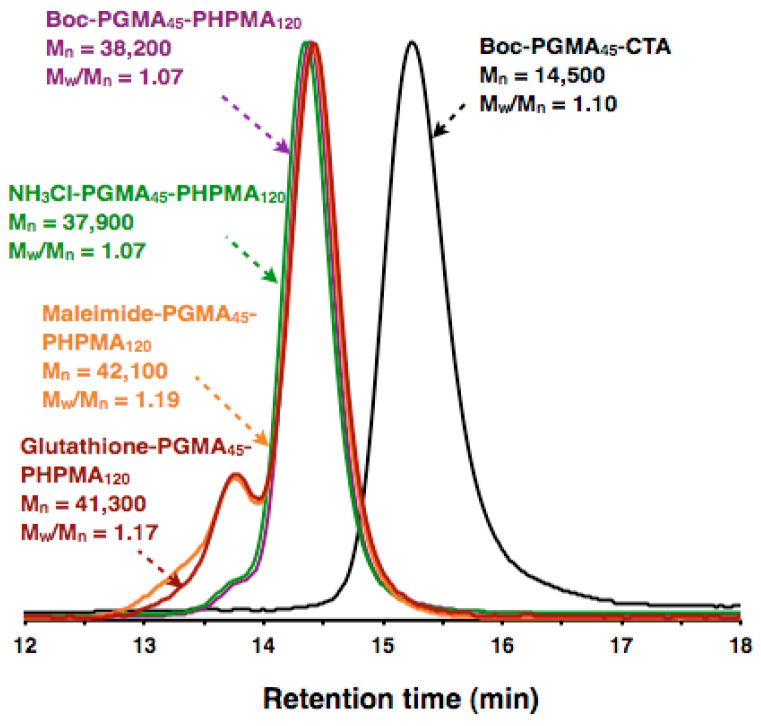
Comparison of DMF GPC traces of *t-Boc*-PGMA_45_ macro CTA and PGMA_45_-PHPMA_120_ copolymers with *t*-*Boc*, primary amine, maleimide, and L-glutathione monofunctionalities.

**Figure 4:**
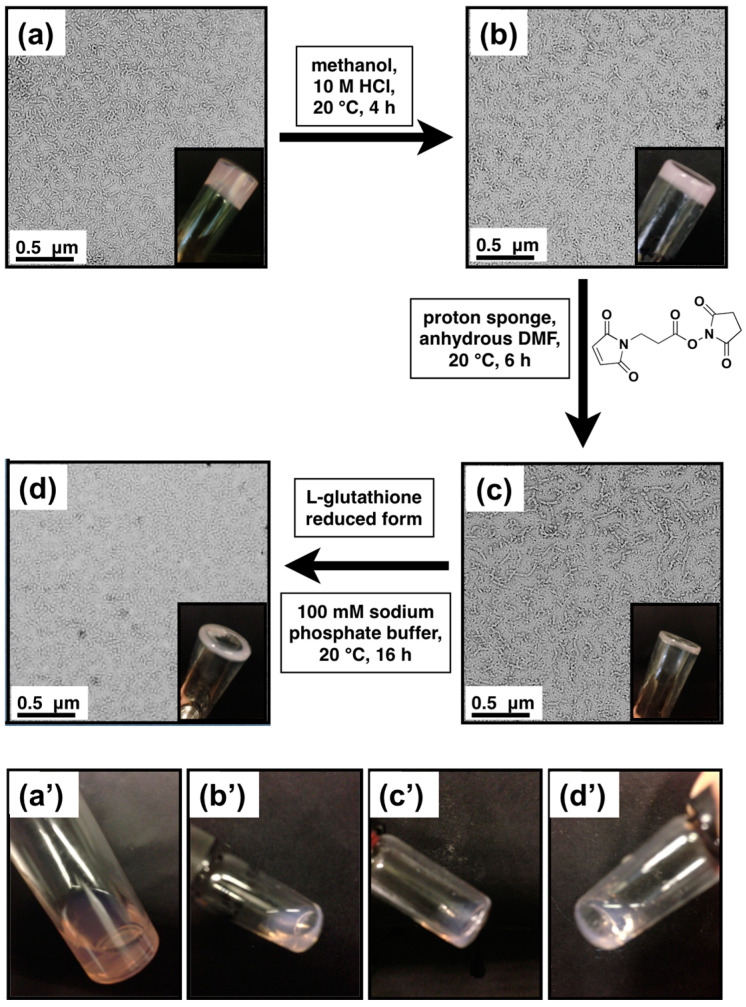
TEM and digital images of (**a**) *t-Boc*-PGMA_45_-PHPMA_120_, (**b**) *NH_3_Cl*-PGMA_45_-PHPMA_120_, (**c**) *Mal*-PGMA_45_-PHPMA_120_, and (**d**) *GSH*-PGMA_45_-PHPMA_120_ copolymer hydrogels (10 *w*/*v*%, PBS 100 mM, pH 7.4), confirmed the maintenance of worm-like morphologies at neutral pH at room temperature before and after post-modification (upper); the digital images of (**a’**) *t-Boc*-PGMA_45_-PHPMA_120_, (**b’**) *NH_3_Cl*-PGMA_45_-PHPMA_120_, (**c’**) *Mal*-PGMA_45_-PHPMA_120_, and (**d’**) *GSH*-PGMA_45_-PHPMA_120_ copolymers (10 *w*/*v*%) in PBS (100 mM, pH 7.4) became liquid after a few minutes stored in the fridge (bottom).

To obtain the block copolymers with primary amine terminals and giving worm-like morphologies under TEM, the RAFT aqueous dispersion polymerization of HPMA was conducted at 10% *w*/*v* in PBS (100 mM, pH 7.4) at 70 °C. The polymerization targeted a series of mean DP for PHPMA block from 100 to 150 [22]. ^1^H-NMR studies indicated more than 99% HPMA conversions within 2 h in 00 all cases. Additionally, comparison of the integrated intensities of the aromatic signals at δ 7.3–8.0 ppm to that of the methyl protons in *t*-*Boc* at δ 1.47 ppm indicated that no degradation of the dithiol ester of the CTA end group happened during the course of polymerization (Figure 2a and Figure S4a in ESI). The DMF GPC studies of these copolymer products indicated a relatively narrow polydispersity (M_w_/M_n_ < 1.10) (Figure 3). However, a small high molecular weight shoulder due to the impurity of HPMA monomer (<0.30 mol% dimethacrylate) was observed [44,45]. The formation of free-standing gels at room temperature was an initial selection criteria indictive of worm-like morphology, which was further confirmed by the TEM images (Figure 4).

To deprotect primary amine functionality, *t*-*Boc* was removed according to the previous report [14,15,46] with some modification. Briefly, *t-Boc*-PGMA_45_-PHPMA_120_ solids were completely dissolved in methanol to make sure all the *t*-*Boc* end groups were accessible for the added hydrochloride acid (10 M) (methanol/HCl = 1:0.5, *v*/*v*) and kept stirring at room temperature for 4 h. The successful generation of primary amine was confirmed by the disappearance of methyl signals on *t*-*Boc* at δ 1.47 ppm and the appearance of a new triplet signal at δ 2.98 ppm assigned to the two methylene protons adjacent to the primary amine in ^1^H-NMR spectrum (Figure 2b and Figure S4b in ESI). Comparing the integrated intensities of this new signal to the aromatic signals attributed to the CTA end indicated no degradation during this process. This was confirmed again by DMF GPC analysis that the molecular weights of PGMA_45_-PHPMA_120_ either with *t*-*Boc* terminals or primary amine terminals were similar, and the polydispersity maintained relatively narrow (M_w_/M_n_ = 1.07) before and after deprotection (Figure 3). The purified *NH_3_Cl*-PGMA_45_-PHPMA_120_ solids were re-dispersed in PBS (100 mM, pH 7.4) at 10% *w*/*v* at room temperature, and observed to become pink free-standing gels after a few minutes (Figure 4). The TEM images indicated that there was no significant change in the worm-like morphology as the effect of the amine generation (Figure 4).

To reduce the surface functionality density of PGMA_45_-PHPMA_120_ worms, HPMA was polymerized via aqueous RAFT dispersion. The polymerization involved a binary mixture of *t-Boc*-PGMA_45_ (M_n_ = 14,500, M_w_/M_n_ = 1.10) and non-functionalized PGMA_42_ (M_n_ = 13,700, M_w_/M_n_ = 1.23) (feeding molar ratio of [*t-Boc*-PGMA_45_]/[PGMA_42_]/[HPMA] = 0.5:0.5:100) macro-CTA under the same condition as described above. Again, the ^1^H-NMR spectrum suggested that the HPMA polymerization was complete within 2 h. The actual molar ratio of [*t-Boc*-PGMA_45_]/[PGMA_42_] was 0.44 to 0.56, which can be calculated from comparison of the integrated intensities of the methyl signals on *t*-*Boc* to those of aromatic signals (Figure S5 in ESI). The primary amine group was then generated within the binary mixed copolymer using the same method as described above. However, the new triplet signal at δ 2.98 ppm assigned to the two methylene protons adjacent to the primary amine was not observed due to the low concentration resulting from the reduced surface density of the functionalities. Additionally, the similar DMF GPC curves of (0.44 *t-Boc*-PGMA_45_ + 0.56 PGMA_42_)-PHPMA_100_ and (0.44 *NH_3_Cl*-PGMA_45_ + 0.56 PGMA_42_)-PHPMA_100_ with relative narrow polydispersity (Figure S6 in ESI) confirmed no degradation happened all through these experiments.

### 3.4. Synthesis of Mal-PGMA_45_-PHPMA_120_ with Worm-Like Morphology

The synthesis of PGMA_45_-PHPMA_120_ copolymers with monofunctionalized maleimide terminals followed a previously reported procedure [38]. The copolymer *NH_3_Cl*-PGMA_45_-PHPMA_120_ solids synthesized were dissolved in anhydrous DMF solution, which serves as an effective aprotic solvent capable of dissolving both blocks. The proton sponge used to deprotonate primary amine from its protonated form and reagent MPA-NHS were dissolved in anhydrous DMF together, and then dropwise added into the above copolymer DMF solution. The molar ratios of primary amine on *NH_3_Cl*-PGMA_45_-PHPMA_120_, MPA-NHS, and proton sponge were 1.0 to 1.5 to 5.0 to achieve a high degree of maleimide functionalization. The ^1^H-NMR spectrum confirmed the successful conjugation by showing the disappearance of the methylene proton signal at δ 2.98 ppm next to primary amine and the appearance of a new signal at δ 6.88 ppm assigned to the two protons of maleimide (Figure 2c and Figure S4c in ESI). Comparison of the integrated intensities of the aromatic signals at δ 7.3–8.0 ppm to that of the two proton signals of maleimide at δ 6.88 ppm indicated almost 100% degree of maleimide functionalization. The DMF GPC analysis of the functionalized copolymer revealed a relatively narrow polydispersity (M_w_/M_n_ = 1.19); however, broader than that of its precursor and a prominent high molecular weight shoulder was observed, indicating the existence of side reactions (Figure 3). Importantly, the TEM images indicated worm-like morphologies for the copolymers after maleimide functionalization (Figure 4).

### 3.5. Synthesis of GSH-PGMA_45_-PHPMA_120_ with Worm-like Morphology

After the maleimide monofunctionalized copolymer worms were successfully synthesized, the work moved to study the reactivity of maleimide terminals and thus their potential ability to conjugate with thiol-containing peptides. Therefore, the reaction between the maleimide functionality and GSH as a model tripeptide in PBS (100 mM, pH 7.4) by Michael addition was performed at room temperature according to the previous reports [38,47]. The products were dialyzed with de-ionized water to remove the free GSH molecule and freeze-dried. The ^1^H-NMR spectrum indicated that the two proton signals at δ 6.88 ppm assigned to the maleimide group disappeared, confirming the success of the Michael addition reacting with thiol group in L-glutathione (Figure 2d and Figure S4d in ESI). However, the new signal at around δ 3.50 ppm belonging to the –CH_2_CH(NH_2_)COOH proton [47] was not observed in d-Methanol. This might be attributed to the low concentration of the reference proton in the solution and/or the overlapping of this signal with unclarified random signals at δ 2.50 to 3.50 ppm. However, the aromatic signals supported the maintenance of the polymer structure, and therefore the successful conjugation of GSH on the copolymer. Additionally, the DMF GPC analysis of PGMA_45_-PHPMA_120_ copolymer with different terminals showed that the GPC trace of *GSH*-PGMA_45_-PHPMA_120_ with the retention time was similar to that of its precursor (*Mal*-PGMA_45_-PHPMA_120_), both of which gave a shoulder at high molecular weight side (Figure 3). Considering this observation with the ^1^H-NMR data together, it therefore came to the conclusion that the disappearance of the two proton signals of maleimide was due to the successful conjugation through Michael addition between the double bonds on maleimide and the thiol on GSH. Importantly, the DMF GPC data also show that the maximum refractive intensities were observed at almost the same retention time position in all copolymer cases with relatively narrow polydispersity, indicating no significant polymer degradation or cross linking all through these experiments.

Particularly, it was also observed that all the monofunctionalized PGMA_45_-PHPMA_120_ copolymers were able to be re-dispersed in PBS (100 mM, pH 7.4) and form free-standing gels at room temperature. However, it became fluent liquid after being stored in a fridge for a few minutes (Figure 4), indicating the modification of polymer terminals did not affect the thermo-responsive behavior of PGMA_45_-PHPMA_120_ copolymers.

### 3.6. Zeta-Potential vs. pH Measurements

**Figure 5:**
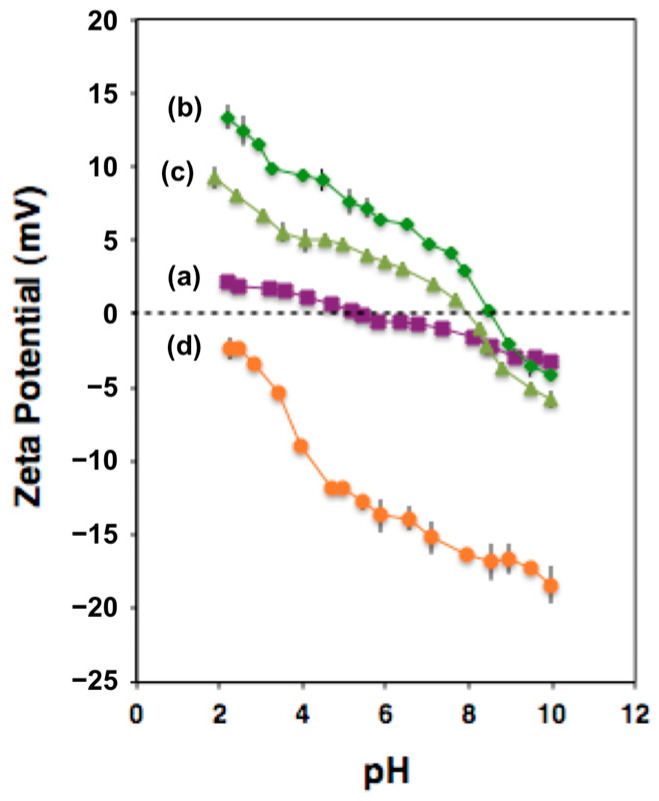
The zeta potential traces of (**a**) *t-Boc*-PGMA_45_-PHPMA_120_, (**b**) *NH_3_Cl*-PGMA_45_-PHPMA_120_, (**c**) (0.44 *NH_3_Cl*-PGMA_45_ + 0.56 PGMA_42_)-PHPMA_100_, and (**d**) *Mal*-PGMA_45_-PHPMA_120_ worms measured at a range of pH from 2 to 10.

The surface charges (zeta potential) of PGMA-PHPMA worms with *t*-*Boc* and primary amine terminals, 44% primary amine terminals [(0.44 *NH_3_Cl*-PGMA_45_ + 0.56 PGMA_42_)-PHPMA_100_], and also maleimide terminals (2 mg/mL) in potassium chloride (KCl) solution (1 mM) at a wide range of pH from 2 to 10 were measured by DLS analysis at 25 °C (Figure 5). The zeta potentials of *t*-*Boc*-PGMA_45_-PHPMA_120_, *NH_3_Cl*-PGMA_45_-PHPMA_120_, (0.44 *NH_3_Cl*-PGMA_45_ + 0.56 PGMA_42_)-PHPMA_100_, and *Mal*-PGMA_45_-PHPMA_120_ worms were found to be 2.2, 13.4, 9.2, and −2.3 mV at approximate pH 2, respectively. The positive surface charge observed for *NH_3_Cl*-PGMA_45_-PHPMA_120_ and (0.44 *NH_3_Cl*-PGMA_45_ + 0.56 PGMA_42_)-PHPMA_100_ were anticipated due to the protonation of the free primary amines at a low pH. The binary mixed (0.44 *NH_3_Cl*-PGMA_45_ + 0.56 PGMA_42_)-PHPMA_100_ giving slightly lower surface charge values than *NH_3_Cl*-PGMA_45_-PHPMA_120_ worms all through the experiment was attributed to the reduced surface density of primary amines on worm surface. The surface charge values of *t-Boc*-PGMA_45_-PHPMA_120_ worms were negligibly small in the experimental pH range as expected. *Mal*-PGMA_45_-PHPMA_120_ worms gave a negative surface charge at the pH range applied possibly due to the hydrolysis of maleimide and the production of maleic acid [48]. Overall, the zeta potential traces of all these four worms decreased with increasing the pH of the testing solution. The isoelectric points (IEPs) of *t-Boc*-PGMA_45_-PHPMA_120_, (0.44 *NH_3_Cl*-PGMA_45_ + 0.56 PGMA_42_)-PHPMA_100_, and *NH_3_Cl*-PGMA_45_-PHPMA_120_ were determined to be 5.3, 8.0, and 8.6, respectively, which were in accord to the surface density of the amines. Additionally, the differences of surface charge among these copolymer worms confirmed the successful monofunctionalization to the end of the copolymer.

## 4. Conclusions

A novel RAFT CTA with *t*-*Boc* group was successfully synthesized with high yields via an optimized process. PGMA_45_-PHPMA_120_ block copolymer was RAFT dispersion polymerized using *t-Boc*-PGMA_45_ macro-CTA, indicating a good control. The precisely targeted worm-like nano-objects, exhibited as free-standing hydrogels at room temperature, were obtained by adjusting the HPMA chain length. PGMA_45_-PHPMA_120_ diblock copolymers with primary amine, maleimide, and reduced L-glutathione monofunctionalities were prepared using the post-polymerization modification method and characterized with ^1^H-NMR, GPC, DLS, and TEM. These modifications had no significant effect on the nano-morphologies as judged by TEM. Since the reversible thermo-responsive behavior of PGMA_45_-PHPMA_120_ worms was still observed in all cases, it is expected that these facile sterilizable hydrogels with varied functionalities on the surface potentially offer attractive applications in biomedical areas, which will be explored in future work.

## Data Availability

Data are contained within the article and supplementary materials.

## Supplementary Materials

The following supporting information can be downloaded at: https://www.mdpi.com/article/10.3390/polym15234590/s1, Figure S1: (**a**) ^1^H-NMR (400 Hz, CDCl_3_), (**b**) ^13^C-NMR (400 Hz, CDCl_3_) and (**c**) HMQC-NMR (400 Hz, CDCl_3_) recorded for 5-(2-(*tert*-butoxycarbonylamino)ethylamino)-2-cyano-5-oxopentan-2-yl benzo-dithioate CTA; Figure S2: Mass spectrum of t-Boc CPDB CTA. ESI-MS: *m*/*z* (MH^+^, 100%) 422, and charged fragments generated from the electron ionization; Figure S3: DMF GPC curves indicating the molecular weight evolution with elution time for the RAFT copolymerization of HPMA at 70 °C using *t-Boc* PGMA_45_ as macro-CTA; Figure S4: ^13^C-NMR (600 Hz, d-methanol) spectra recorded for (**a**) *t-Boc* protected PGMA_45_-PHPMA_120_ copolymer RAFT polymerized by using *t-Boc*-PGMA_45_ macro-CTA in PBS (100 mM, pH 7.4), (**b**) NH_3_Cl-PGMA_45_-PHPMA_120_ generated with HCl (10 M) in methanol, (**c**) Mal-PGMA_45_-PHPMA_120_ copolymer synthesized by reacting NH_3_Cl-PGMA_45_-PHPMA_120_ with MPA-NHS in anhydrous DMF at room temperature, and (**d**) GSH conjugated PGMA_45_-PHPMA_120_ copolymer synthesized in 100 mM PBS (100 mM, pH 7.4) at room temperature; Figure S5: ^1^H-NMR spectra of (**a**) (0.44 *t-Boc*-PGMA_45_ + 0.56 PGMA_42_)–PHPMA_100_ and (**b**) (0.44 NH_3_Cl-PGMA_45_ + 0.56 PGMA_42_)-PHPMA_100_ copolymers synthesized; Figure S6: Comparison of DMF GPC traces of *t-Boc*-PGMA_45_ and PGMA_42_ macro-CTA, (0.44 *t-Boc*-PGMA_45_ + 0.56 PGMA_42_)-PHPMA_100_ and (0.44 NH_3_Cl-PGMA_45_ + 0.56 PGMA_42_)-PHPMA_100_ copolymer worms synthesized.
