# Supplementary material for: Synthesis of Thermo-Responsive Monofunctionalized Diblock Copolymer Worms
Source: Polymers (Basel). 2023 Nov 30;15(23):4590. doi: 10.3390/polym15234590 (PMC10708438; doi:10.3390/polym15234590)
Supplement: Supplementary file 1 [file polymers-15-04590-s001.zip › polymers-2720505-supplementary.pdf]

Electronic Supplementary Information

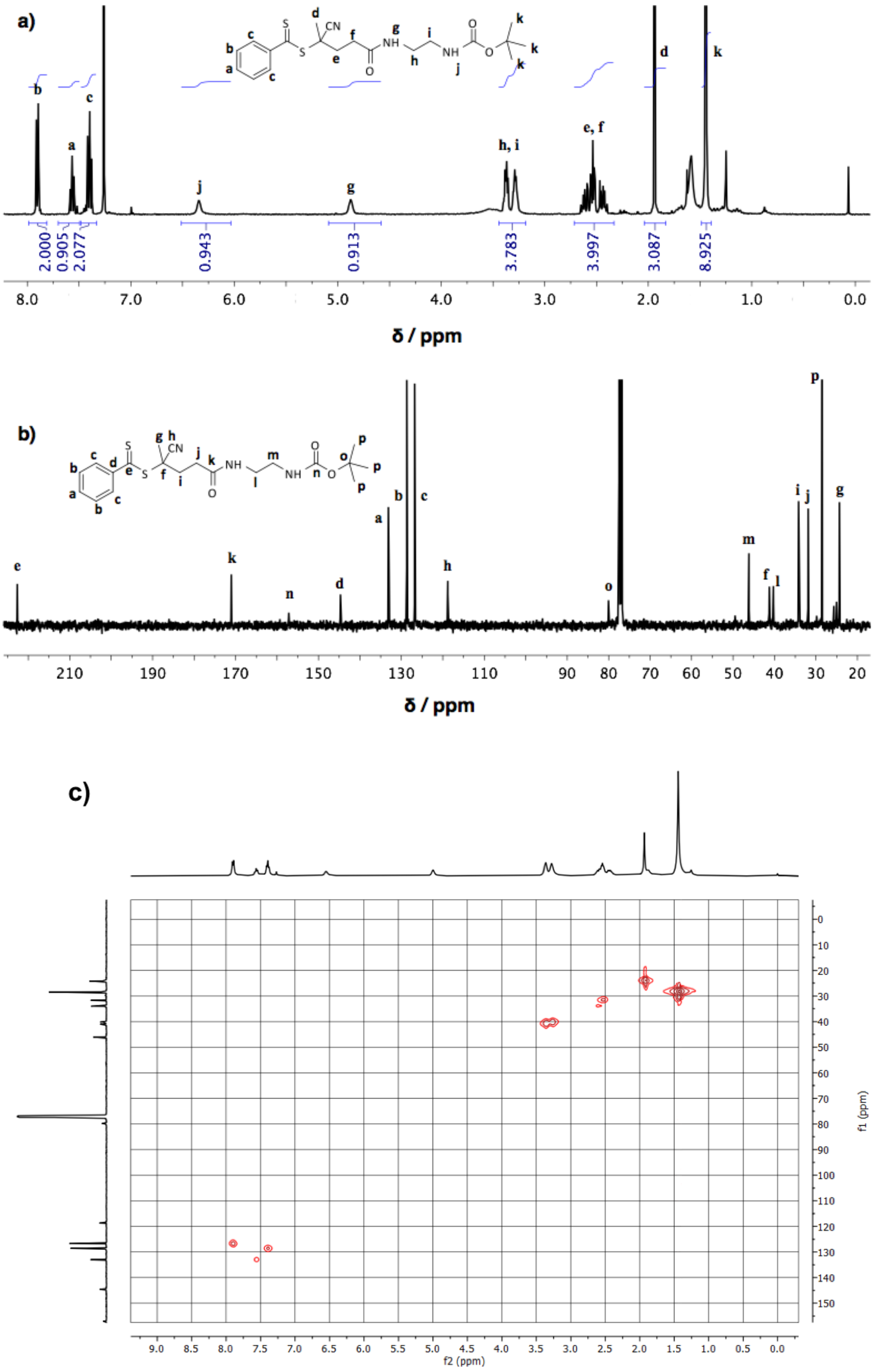

Figure S1. a) ^{1}H-NMR (400 Hz, $CDCl_3$), b) ^{13}C-NMR (400 Hz, $CDCl_3$) and c) HMQC-NMR (400 Hz, $CDCl_3$) recorded for 5-(2-(*tert*-butoxycarbonylamino)ethylamino)-2-cyano-5-oxopentan-2-yl benzo-dithioate CTA

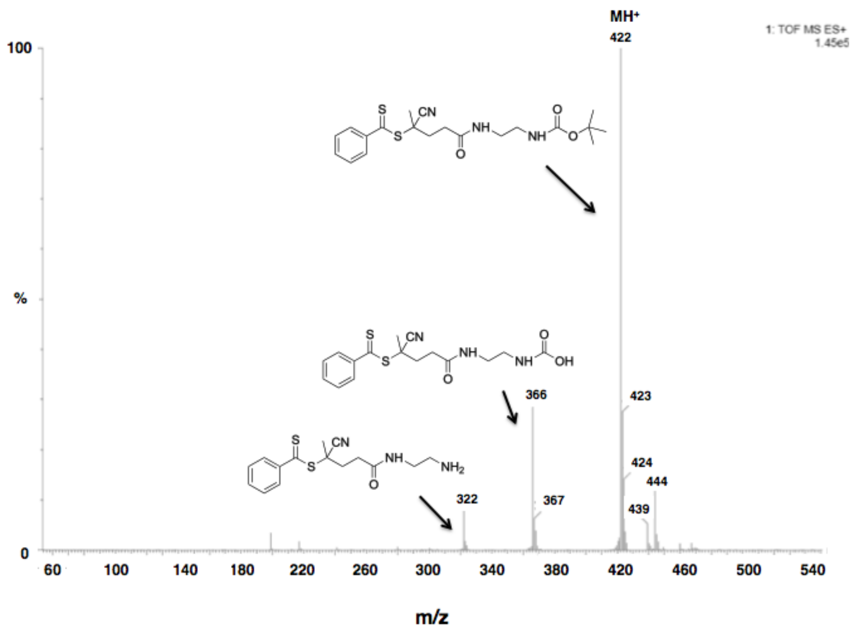

Figure S2. Mass spectrum of *t-Boc* CPDB CTA. ESI-MS: m/z (MH^+, 100%) 422, and charged fragments generated from the electron ionization.

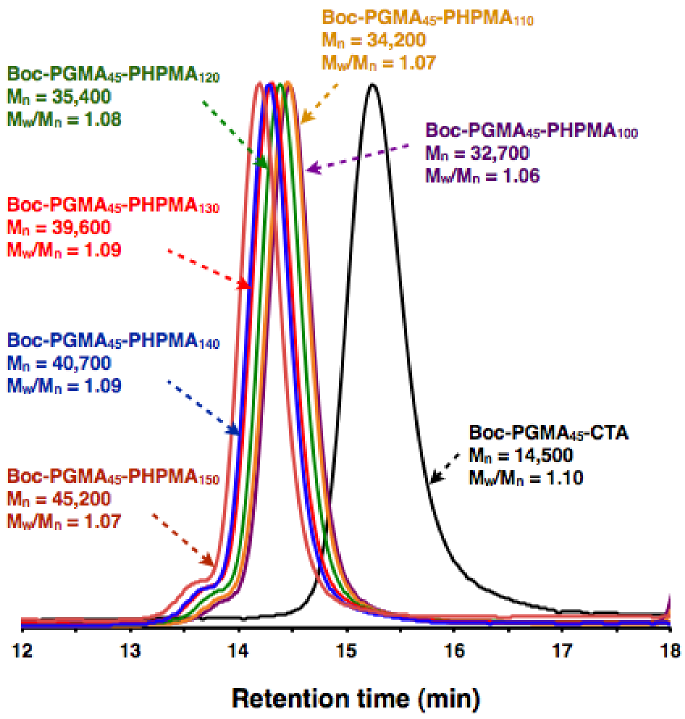

Figure S3. DMF GPC curves indicating the molecular weight evolution with elution time for the RAFT copolymerization of HPMA at 70 °C using *t-Boc* $PGMA_{45}$ as macro-CTA

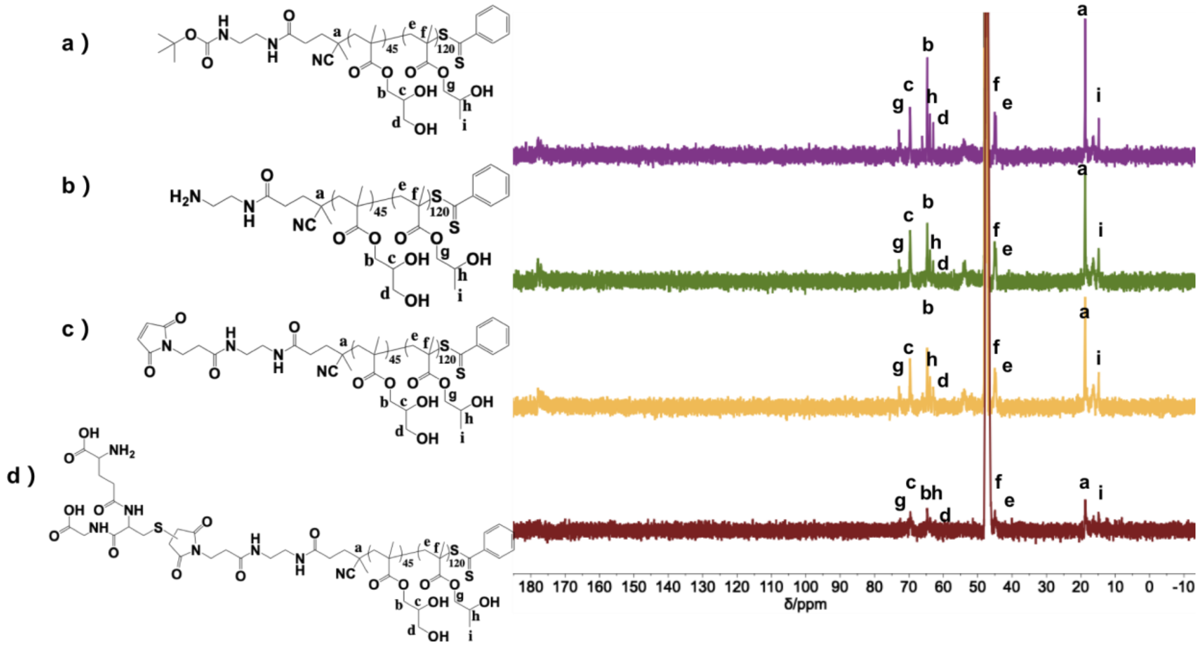

Figure S4. ^{13}C-NMR (600 Hz, d-methanol) spectra recorded for a) *t*-Boc protected PGMA$_{45}$-PHPMA$_{120}$ copolymer RAFT polymerized by using *t-Boc*-PGMA$_{45}$ macro-CTA in PBS (100 mM, pH 7.4), b) *NH$_3$Cl*-PGMA$_{45}$-PHPMA$_{120}$ generated with HCl (10 M) in methanol, c) *Mal*-PGMA$_{45}$-PHPMA$_{120}$ copolymer synthesized by reacting *NH$_3$Cl*-PGMA$_{45}$-PHPMA$_{120}$ with MPA-NHS in anhydrous DMF at room temperature, and d) GSH conjugated PGMA$_{45}$-PHPMA$_{120}$ copolymer synthesized in 100 mM PBS (100 mM, pH 7.4) at room temperature.

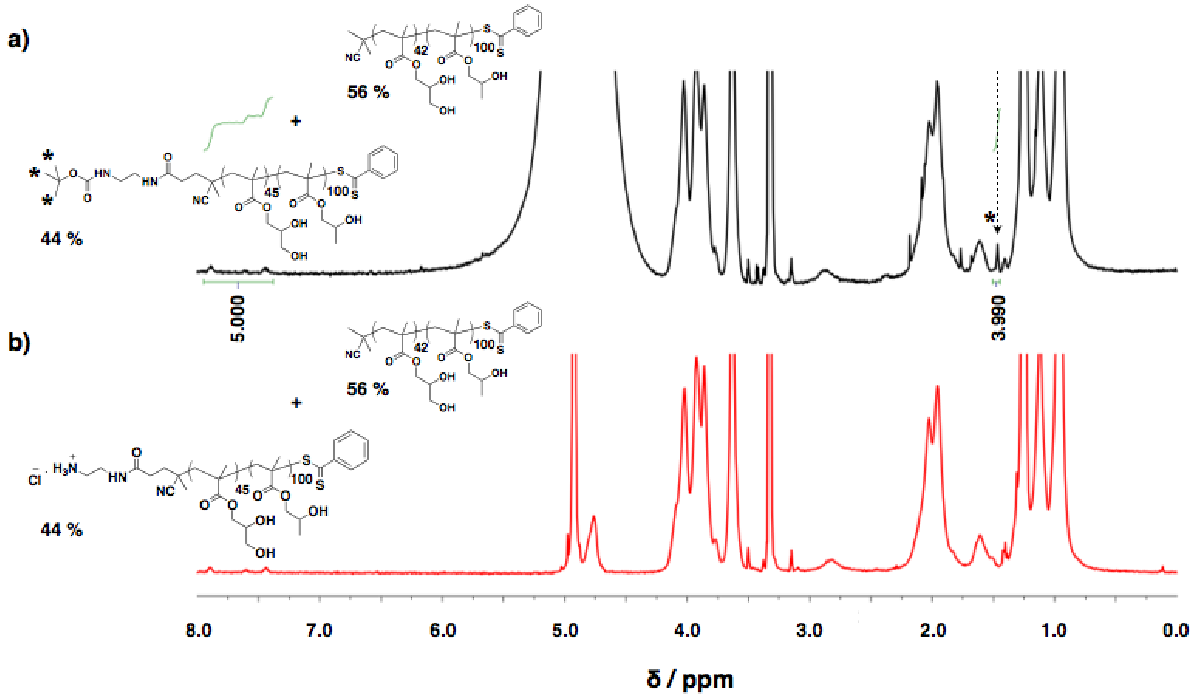

Figure S5. ^{1}H-NMR spectra of a) (0.44*t-Boc*-PGMA$_{45}$ + 0.56PGMA$_{42}$)–PHPMA$_{100}$ and b) (0.44*NH$_3$Cl*-PGMA$_{45}$ + 0.56PGMA$_{42}$)-PHPMA$_{100}$ copolymers synthesized

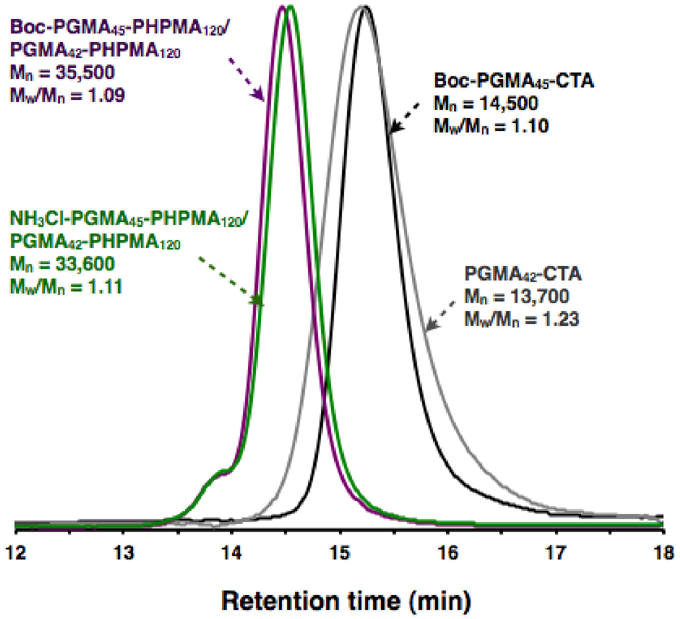

Figure S6. Comparison of DMF GPC traces of *t-Boc*-$PGMA_{45}$ and $PGMA_{42}$ macro-CTA, (o.44*t-Boc*-$PGMA_{45}$ + 0.56$PGMA_{42}$)-$PHPMA_{100}$ and (0.44*NH_3Cl*-$PGMA_{45}$ + 0.56$PGMA_{42}$)-$PHPMA_{100}$ copolymer worms synthesized
